# Lipid Emulsion Mitigates the Cardiotoxic Effects of Labetalol in Rat Cardiomyoblasts

**DOI:** 10.3390/cells14030187

**Published:** 2025-01-26

**Authors:** Gyujin Sim, Seong-Ho Ok, Soo Hee Lee, Kyeong-Eon Park, Seunghyeon Park, Ju-Tae Sohn

**Affiliations:** 1 Department of Anesthesiology and Pain Medicine, Gyeongsang National University Hospital, Jinju-si 52727, Republic of Korea; 2 Institute of Medical Science, Gyeongsang National University, Jinju-si 52727, Republic of Korea; 3 Department of Anesthesiology and Pain Medicine, Gyeongsang National University Changwon Hospital, Changwon-si 51472, Republic of Korea; 4 Department of Anesthesiology and Pain Medicine, Gyeongsang National University College of Medicine, Jinju-si 52727, Republic of Korea; 5 Department of Anesthesiology and Pain Medicine, Gyeongsang National University College of Medicine, Gyeongsang National University Hospital, Jinju-si 52727, Republic of Korea

**Keywords:** labetalol, lipid emulsion, toxicity, PI3K, Akt, GSK-3β, cardiomyoblast

## Abstract

Lipid emulsion has recently emerged as an effective agent for improving the cardiotoxicity of highly lipophilic drugs. However, its effect on cardiotoxicity induced by labetalol, a nonselective beta-blocker, remains unknown. In this study, we investigated the effects of lipid emulsion on the cardiotoxicity of labetalol in rat cardiomyoblasts and tried to decipher the underlying mechanisms. The effects of lipid emulsion on labetalol-induced changes in cell viability, expression of Bax/Bcl-2, cleaved caspase-3, and cleaved caspase-9, and phosphorylation of GSK-3β, Akt, and PI3K were examined. Lipid emulsion inhibited labetalol-induced decrease in cell viability, whereas LY294002, MK2206, and SB216763, the inhibitors of phosphoinositide 3-kinase (PI3K), Akt, glycogen synthase kinase-3β (GSK-3β), respectively, partially attenuated this restoration of cell viability. Lipid emulsion reversed the increase in expression of cleaved caspase-3, cleaved caspase-9, and Bax/Bcl-2 and decrease in the phosphorylation of GSK-3β, Akt, and PI3K by labetalol. Lipid emulsion and cyclosporin, a mitochondrial permeability transition pore (MPTP) inhibitor, reduced the labetalol-induced increase in the number of TUNEL-positive cells and promoted late-stage apoptosis. Overall, lipid emulsion inhibited apoptotic cell death caused by labetalol toxicity via the inhibition of intrinsic apoptotic pathway and MPTP in rat cardiomyoblasts, which appears to involve PI3K, Akt, and GSK-3β signaling pathways.

## 1. Introduction

Labetalol is an alpha-1 and nonselective beta-adrenoceptor blocker used for treating essential hypertension, renal hypertension, pheochromocytoma, and hypertensive emergency [1]. The ratio of beta-adrenoceptor blockade to alpha-1 adrenoceptor blockade by labetalol is 3:1 [1]. Beta-1 adrenoceptor stimulation of cardiac myocytes induces calcium influx via the opening of calcium channels, which leads to the activation of adenylate cyclase and cyclic adenosine monophosphate formation [2]. The increased cyclic adenosine monophosphate levels induce the opening of the L-type calcium channels and calcium influx, leading to positive inotropic and chronotropic effects [2]. Beta-blocker or calcium channel blocker toxicity in the heart ultimately inhibits calcium influx [2]. Toxic doses of beta-blockers severely inhibit calcium influx, leading to bradycardia, hypotension, conduction abnormalities, and shock [2]. The pharmacologic treatment of high-dose beta-blocker toxicity includes calcium, glucagon, catecholamine (adrenoceptor agonists), insulin-euglycemia, and sodium bicarbonate [2].

Lipid emulsion, initially created for parenteral nutrition, has recently emerged as an effective agent for improving cardiovascular collapse triggered by toxic doses of highly lipophilic (log P [octanol/water partition coefficient]: >2) non-local anesthetic drugs, including those used in cardiovascular and neuropsychiatric treatments [3]. Additionally, lipid emulsion reportedly alleviates cardiotoxicity evoked by toxic doses of propranolol, a highly lipid-soluble beta-blocker (log P: 3.48), in pediatric and adult patients [4,5]. Moreover, lipid emulsion has been used as an adjuvant drug for treating hemodynamic collapse triggered by toxic doses of labetalol and amlodipine [6]. Reduction of cardiac toxicity caused by local anesthetic bupivacaine and calcium channel blocker verapamil or cardiac ischemic reperfusion injury by lipid emulsion has been suggested to be associated with the pathway involving phosphorylation of phosphoinositide-3 kinase (PI3K), Akt, and glycogen synthase kinase-3β (GSK-3β) [7,8,9]. The lipid shuttle mechanism, a widely accepted explanation for the role of lipid emulsion as an adjunct in drug toxicity treatment, suggests that lipid emulsion sequesters highly lipophilic drugs, for example, bupivacaine, from organs, such as the brain and heart [3]. This lipid-bound drug is then carried to the liver, muscle, and fat tissue, where it is detoxified and stored [3]. Lipid emulsion also lowers the concentration of labetalol, which is a highly lipid-soluble drug (log P = 2.7) [10,11]. However, the effect of lipid emulsion on the cardiotoxicity of labetalol remains unknown.

Based on previous reports and the high lipid solubility of labetalol, we hypothesized that lipid emulsion would reduce the cardiotoxicity induced by toxic doses of labetalol in cardiomyoblasts [3,6,10,11]. In this in vitro study, we aimed to evaluate the impact of lipid emulsion on cardiotoxicity caused by a toxic dose of labetalol in H9c2 rat cardiomyoblasts and to decipher the underlying mechanism, particularly in relation to the phosphorylation of PI3K, Akt, and GSK-3β.

## 2. Materials and Methods

### 2.1. Materials

All commercially available drugs of greatest purity were utilized in the experiments. Labetalol, LY294002, cyclosporin, and anti-β-actin were procured from Sigma-Aldrich (St. Louis, MO, USA). SB216763 was acquired from Tocris Bioscience (Bristol, UK). Intralipid (20%) was procured from Fresenius Kabi (Uppsala, Sweden). Intralipid 20% (molecular weight 871 g/mol) from soybean, which was used in this study, comprises linoleic acid (53%), palmitic acid (11%), oleic acid (24%), stearic acid (4%), and alpha-linolenic acid (8%) [12,13]. Antibodies against cleaved caspase-3, Bax, Akt, phospho-Akt, PI3K, phospho-PI3K, GSK-3β, and phospho-GSK-3β were acquired from Cell Signaling Technology (Beverly, MA, USA). The anti-cleaved caspase-8 antibody was procured from Novus Biologicals (Centennial, CO, USA). The anti-Bcl-2 antibody was sourced from Santa Cruz Biotechnology (Santa Cruz, CA, USA), and MK2206 was procured from Cayman Chemical (Ann Arbor, MI, USA). The anti-cleaved caspase-9 antibody was purchased from Enzo Biochem Inc. (Farmingdale, NY, USA).

### 2.2. Cell Culture

**Figure 1:**
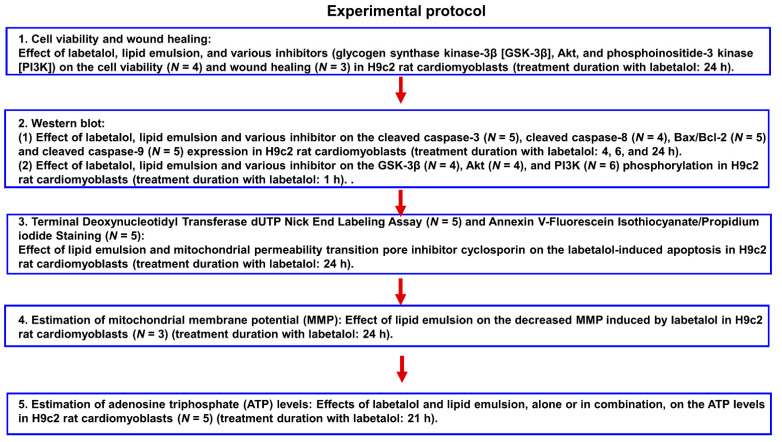
Experimental protocol. *N* indicates the number of independent experiments.

The H9c2 rat cardiomyoblasts were procured from the American Type Culture Collection (Rockville, MD, USA). These cells were cultured in high-glucose Dulbecco’s modified Eagle medium (DMEM) (Gibco, Life Technologies, Grand Island, NY, USA), supplemented with 1% penicillin/streptomycin and 10% fetal bovine serum (both sourced from Gibco). The cells were maintained at 37 °C in a 5% CO_2_ environment, as previously reported by Lee et al. [14]. Prior to drug administration, cells were subjected to a 16-h preincubation in serum-free DMEM. The experimental protocols are described in Figure 1.

### 2.3. Cell Viability

Cell viability was evaluated using the Cell Counting Kit-8 (CCK-8; Dojindo Molecular Technologies, Kumamoto, Japan), as specified by the manufacturer and outlined by Lee et al. [14]. In brief, each well of the 24-well plates was seeded with 3 × 10^4^ H9c2 cells. They were treated for 24 h with different concentrations of labetalol, ranging from 3 × 10^−6^ M to 3 × 10^−4^ M. Moreover, because 50% of cells were viable when treated with 2.5 × 10^−4^ M labetalol, this concentration was selected for examining the effects of lipid emulsion and various inhibitors, alone or in combination, on the labetalol-induced decrease in cell viability. To examine the role of lipid emulsion (Intralipid) in mitigating the decrease in cell viability triggered by a toxic dose (2.5 × 10^−4^ M) of labetalol (toxic concentration of plasma labetalol [1 μg/mL]: 1.94 × 10^−6^ M), the cells were treated with 2.5 × 10^−4^ M labetalol alone for 24 h, lipid emulsion (0.1, 0.2, 0.3, 0.5, 0.7, and 1%) for 1 h followed by 2.5 × 10^−4^ M labetalol for 24 h, or lipid emulsion (0.1, 0.2, 0.3, 0.5, 0.7, and 1%) alone for 25 h [15]. Furthermore, to examine the potential role of the PI3K, Akt, and GSK-3β pathways in the protective effect of lipid emulsion on cell viability against toxic labetalol concentrations, cells were subjected to the following treatments: 2.5 × 10^−4^ M labetalol alone for 24 h; 1% lipid emulsion for 1 h followed by 2.5 × 10^−4^ M labetalol for 24 h; PI3K inhibitor (10^−6^ M LY294002), GSK-3β inhibitor (5 × 10^−6^ M SB216763), or Akt inhibitor (10^−7^ M MK 2206) for 1 h, followed by 1% lipid emulsion for 1 h and 2.5 × 10^−4^ M labetalol for 24 h; lipid emulsion alone for 25 h; or inhibitor alone for 26 h [7]. The concentrations of inhibitors used in this experiment were chosen by referring to a previous study [7]. The cells were treated with 200 μL of DMEM containing 10% CCK-8 solution and incubated for 3 h at 37 °C in a 5% CO_2_ environment. The VersaMax microplate reader (Molecular Devices, Sunnyvale, CA, USA) was used to measure the absorbance at 450 nm. Cell viability was estimated as follows:

Cell viability (%) = (optical density of the drug treatment group/optical density of the control group) × 100

Four independent experiments were performed.

### 2.4. Wound-Healing Assay

We performed a wound-healing test on H9c2 cells, following the procedure detailed by Lee et al. [14], to examine the effect of lipid emulsion on cell migration after labetalol administration. In 100 mm culture dishes, H9c2 cells were seeded at a density of 1 × 10^6^ and incubated at 37 °C in an environment with 5% CO_2_. When the cells were 90–100% confluent, the dish surface was scratched with a 200 μL pipette tip. The dishes were then washed with phosphate-buffered saline (PBS), and a complete culture medium was added. These dishes were photographed under a Nikon Eclipse Ti2 microscope (Nikon Co., Tokyo, Japan). The cells were then subjected to the following treatments: 2.5 × 10^−4^ M labetalol alone for 24 h; 1% lipid emulsion for 1 h followed by 2.5 × 10^−4^ M labetalol for 24 h; and 1% lipid emulsion for 25 h without any other treatment. The cells were subsequently photographed under the Nikon Eclipse Ti2 light microscope. The percent wound healing area was calculated using the following formula:

Wound healing area (%) = (Wound area 24 h after scratching/wound area immediately after scratching) × 100

Three independent experiments were performed.

### 2.5. Western Blot Analysis

Western blotting was used to examine the expression levels of cleaved caspase-3, Bax, Bcl-2, cleaved caspase-9, and cleaved caspase-8 proteins, as well as to evaluate the phosphorylation of GSK-3β, Akt, and PI3K in H9c2 cells, as described previously [14]. To evaluate the expression of cleaved caspase-3 and cleaved caspase-9, the cells were subjected to the following treatments: 2.5 × 10^−4^ M labetalol for 24 h; 1% lipid emulsion for 1 h followed by 2.5 × 10^−4^ M labetalol for 24 h; inhibitors (10^−6^ M LY294002 or 5 × 10^−6^ M SB216763) for 1 h followed by 1% lipid emulsion for 1 h and 2.5 × 10^−4^ M labetalol for 24 h; 1% lipid emulsion alone for 25 h; or inhibitor alone for 26 h. To assess the expression of cleaved caspase-8, the cells were treated with 2.5 × 10^−4^ M labetalol for 6 h. The Bax and Bcl-2 expression levels were determined after the treatment of cells for different durations. The cells were treated with 2.5 × 10^−4^ M labetalol for 4 h, 1% lipid emulsion for 1 h followed by 2.5 × 10^−4^ M labetalol for 4 h, or 1% lipid emulsion for 5 h. To detect GSK-3β phosphorylation, the cells were subjected to the following treatments: labetalol (2.5 × 10^−4^ M) alone for 1 h; 1% lipid emulsion for 1 h followed by labetalol (2.5 × 10^−4^ M) for 1 h, SB216763 (5 × 10^−6^ M) for 1 h followed by 1% lipid emulsion for 1 h and then labetalol (2.5 × 10^−4^ M) for 1 h, 1% lipid emulsion alone for 2 h, or SB216763 (5 × 10^−6^ M) alone for 3 h. To evaluate the phosphorylation of Akt and PI3K, the cells were exposed to the following treatments: labetalol (2.5 × 10^−4^ M) alone for 1 h; 1% lipid emulsion for 1 h followed by labetalol (2.5 × 10^−4^ M) for 1 h; inhibitor (10^−5^ M LY294002 or 10^−7^ M MK2206) for 1 h followed by 1% lipid emulsion for 1 h and labetalol (2.5 × 10^−4^ M) for 1 h; 1% lipid emulsion alone for 2 h; and inhibitor (10^−5^ M LY294002 or 10^−7^ M MK2206) alone for 3 h [16]. The concentration of LY294002 used in this experiment was chosen by referring to a previous study [16]. Following the treatments, the cells were lysed in radio-immunoprecipitation assay buffer (Cell Signaling Technology) supplemented with protease and phosphatase inhibitor cocktails (Thermo Fisher Scientific, Rockfield, IL, USA). Protein concentrations were determined with a bicinchoninic acid protein assay kit (Thermo Fisher Scientific). The lysate was heated at 100 °C for 10 min to denature the proteins, which were then separated using 8–14% SDS-PAGE and transferred onto PVDF membranes (Millipore, Bedford, MA, USA). The membranes were blocked with tris-buffered saline with 0.5% Tween-20 (TBST) containing 5% skim milk or bovine serum albumin for 1 h at room temperature (22–27 °C) and then incubated with primary antibodies overnight at 4 °C (anti-cleaved caspase-3 [1:1000], anti-cleaved caspase-8 [1:1000], anti-Bax [1:1000], anti-cleaved caspase-9 [1:1000], anti-Bcl-2 [1:250], anti-GSK-3β [1:1000], anti-phospho-GSK-3β [1:1000], anti-Akt [1:1000], anti-phospho-Akt [1:1000], anti-PI3K [1:1000], anti-phospho-PI3K [1:1000], or anti-β-actin [1:10,000]). After washing with TBST, the membranes were incubated with horseradish peroxidase-conjugated secondary antibodies against rabbit IgG or mouse IgG (1:5000) for 1 h at room temperature. Protein bands were visualized using the Westernbright™ ECL detection kit (Advansta, Menlo Park, CA, USA), and ECL images were acquired with a ChemiDoc™ Touch Imaging System (Bio-Rad Laboratories Inc., Berkeley, CA, USA). Band densities were determined using the ImageJ software (version 1.45s; National Institutes of Health, Bethesda, MD, USA). The relative density of cleaved caspase-3, Bax, Bcl-2, cleaved caspase-9, and cleaved caspase-8 bands were normalized to β-actin levels. The relative density of bands for the phosphorylated proteins was normalized to the band intensity of the total protein. β-actin served as the loading control. Five independent experiments were performed to determine Bax/Bcl-2, cleaved caspase-9, and cleaved caspase-3 expression. Four independent experiments were performed for determining the expression of cleaved caspase-8 and phosphorylation of GSK-3β and Akt. Six independent experiments were performed to determine PI3K phosphorylation.

### 2.6. Terminal Deoxynucleotidyl Transferase dUTP Nick End Labeling (TUNEL) Assay

The TUNEL assay was used to detect apoptotic cells, following the procedures outlined by Lee et al. [14]. The In Situ Cell Death Detection Kit, a TUNEL kit (Roche Applied Science, Indianapolis, IN, USA), was used as directed by the manufacturer. The H9c2 cells (seeded at a density of 1 × 10^5^) were grown on coverslips in 6-well plates and then exposed to different treatments: 2.5 × 10^−4^ M labetalol alone for 24 h; 1% lipid emulsion (Intralipid) for 1 h followed by 2.5 × 10^−4^ M labetalol for 24 h; or 1% lipid emulsion alone for 25 h. The cells were also treated with the mitochondrial permeability transition pore (MPTP) inhibitor cyclosporin (10^−8^ M) for 1 h before adding 2.5 × 10^−4^ M labetalol for 24 h, or cyclosporin (10^−8^ M) alone for 25 h [17]. The cells were counterstained with 4′,6-diamidino-2-phenylindole (DAPI) after the treatment. Fluorescent pictures were captured using a Nikon Eclipse Ti2 fluorescence microscope. The percentage of TUNEL-positive cells was calculated using the following equation:

TUNEL-positive cells (%) = (total number of TUNEL-positive cells/total number of DAPI-stained cells) × 100

Five separate experiments were conducted.

### 2.7. Annexin V-Fluorescein Isothiocyanate/Propidium Iodide (Annexin V-FITC/PI) Staining

Early and late apoptosis was assessed using the FITC Annexin V Apoptosis Detection Kit (Invitrogen-Life Technologies, Carlsbad, CA, USA) in accordance with the manufacturer’s protocol, as described previously by Lee et al. [14]. In brief, each well of a 6-well plate was seeded with a total of 2 × 10^5^ cells. Treatments included 2.5 × 10^−4^ M labetalol alone for 24 h, 1% lipid emulsion (Intralipid) for 1 h, and subsequent exposure to 2.5 × 10^−4^ M labetalol for 24 h or 1% lipid emulsion alone for 25 h. The cells were then collected, washed twice with PBS, and suspended in 1× annexin binding buffer. Thereafter, 5 μL annexin V-FITC and 1 μL PI staining solution (100 μg/mL) were added, and the plates were incubated at room temperature in a darkened environment for 15 min. The cells were treated with 1× annexin binding solution and examined using a BD LSRFortessa X-20 flow cytometer (BD Biosciences, Franklin Lakes, NJ, USA) to calculate the fraction of cells exhibiting apoptotic characteristics. The data were analyzed using the BD Biosciences FACSDiva^TM^ software (version 6.0). Five separate experiments were performed.

### 2.8. Estimation of Mitochondrial Membrane Potential

The JC-1 Mitochondrial Membrane Potential (MMP) Detection Kit (Invitrogen) was employed to evaluate mitochondrial membrane potential, as reported by Lee et al. [14]. H9c2 cells were seeded on cover glasses kept in 6-well plates at a density of 1 × 10^5^ cells per well. The cells were exposed to one of the following three treatments: 2.5 × 10^−4^ M labetalol for 24 h; 1% lipid emulsion (Intralipid) for 1 h followed by 2.5 × 10^−4^ M labetalol for 24 h; or 1% lipid emulsion alone for 25 h. The cells were rinsed with PBS and incubated with the JC-1 dye (10 μg/mL) at 37 °C in the dark for 30 min. They were subsequently washed with PBS. The MMP was determined by comparing the ratio of red to green fluorescence intensity, which was subsequently normalized to the control using a Nikon Eclipse Ti2 fluorescence microscope. MMP was calculated using the following equation:

MMP = ratio of JC-1 red to green fluorescence intensity in the drug-treated group/ratio of JC-1 red to green fluorescence intensity in the control group (1)

Three separate experiments were performed.

### 2.9. Estimation of Adenosine Triphosphate (ATP) Levels

The intracellular ATP content in labetalol-treated H9c2 cells was quantified using a commercially available ATP detection kit (Abcam, Cambridge, MA, USA) following the methodology reported by Lee et al. [14]. A total of 1 × 10^6^ H9c2 cells were seeded in 100 mm culture dishes and cultured at 37 °C in a humidified atmosphere with 5% CO_2_. Thereafter, they were starved by incubating in a serum-free medium. These cells were exposed to either 2.5 × 10^−4^ M labetalol alone for 21 h, 1% lipid emulsion (Intralipid) for 1 h, followed by 2.5 × 10^−4^ M labetalol for 21 h, or 1% lipid emulsion for 22 h. After the treatment, the cells were lysed using an ATP assay buffer and subsequently centrifuged at 13,000× *g* for 5 min at 4 °C. The supernatant was transferred to another tube. The samples and standards were mixed with an ATP reaction mixture and incubated in the dark at room temperature for 30 min. Thereafter, the absorbance at 570 nm was measured with a VersaMax microplate reader to quantify the intracellular ATP levels. A standard curve of ATP was generated for calibration. The experiment was performed five times.

### 2.10. Statistical Analysis

The major objective of this study was to investigate the impact of lipid emulsion and different inhibitors on the reduced cell viability caused by a toxic dosage of labetalol in rat cardiomyoblasts. We used the Kolmogorov–Smirnov test to check the normal distribution of data. One-way analysis of variance (ANOVA) followed by Bonferroni’s multiple comparison test (Prism 5.0 [GraphPad, Inc., San Diego, CA, USA]) was used to examine the effects of labetalol, lipid emulsion, and different inhibitors on cell viability, wound healing area, cleaved caspase-3 and Bax/Bcl-2 expression, and GSK-3β, Akt, and PI3K phosphorylation. The effect of labetalol on the expression of cleaved caspase-8 was evaluated using the Student’s *t*-test. The Kruskal–Wallis and Dunn’s multiple comparison tests were used to examine the effect of labetalol and lipid emulsion on the expression of cleaved caspase-9. The effects of labetalol and lipid emulsion on TUNEL-positive cells, apoptosis, mitochondrial membrane potential, and ATP levels were examined using one-way ANOVA followed by Bonferroni’s multiple comparison test. A value of *p* < 0.05 was considered statistically significant.

## 3. Results

**Figure 2:**
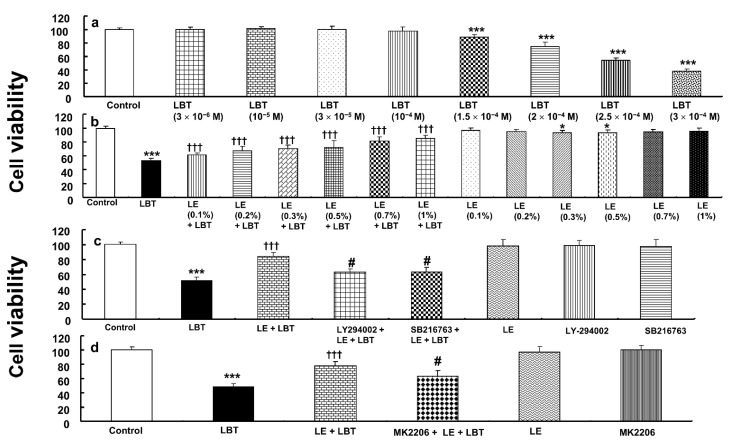
(**a**) Effect of labetalol (LBT) on the viability of H9c2 rat cardiomyoblasts. (**b**–**d**) Effect of lipid emulsion (LE, 1%) and different inhibitors (10^−6^ M LY294002, 5 × 10^−6^ M SB216763, and 10^−7^ M MK2206), either individually or in combination, on the reduction of cardiomyoblast viability induced by LBT (2.5 × 10^−4^ M). Data (*N* = 4) are presented as mean ± SD, with *N* representing the number of separate experiments. * *p* < 0.05 and *** *p* < 0.001 compared to the control. ††† *p* < 0.001 compared to LBT alone. # *p* < 0.001 compared to LE + LBT.

Labetalol (1.5 × 10^−4^ to 3 × 10^−4^ M) decreased the viability of H9c2 rat cardiomyoblasts (*p* < 0.001 compared to control, Figure 2a). However, lipid emulsion (0.1–1%) restored the cell viability reduced by labetalol (2.5 × 10^−4^ M) (*p* < 0.001 compared to labetalol alone, Figure 2b). The PI3K inhibitor LY294002 (10^−6^ M) and the GSK-3β inhibitor SB216763 (5 × 10^−6^ M) partially blocked the effect of lipid emulsion (1%) in reversing the reduction of cell viability caused by labetalol (2.5 × 10^−4^ M) (*p* < 0.001 compared to lipid emulsion plus labetalol, Figure 2c). The Akt inhibitor MK2206 (10^−7^ M) also prevented the reversal of the labetalol (2.5 × 10^−4^ M)-induced decrease in cell viability by the lipid emulsion (1%) (*p* < 0.001 compared to lipid emulsion plus labetalol, Figure 2d).

**Figure 3:**
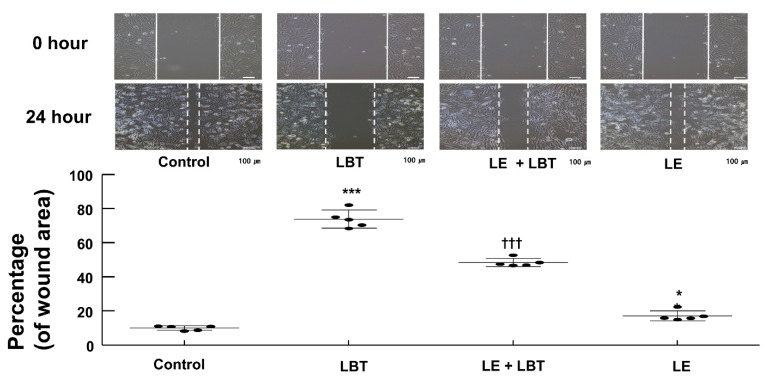
Effect of lipid emulsion (LE, 1%) and labetalol (LBT, 2.5 × 10^−4^ M), used alone or in combination, on wound healing in H9c2 rat cardiomyoblasts, as measured using the scratch assay. The results (*N* = 3) are presented as mean ± SD, with *N* representing the number of separate experiments. * *p* < 0.05 and *** *p* < 0.001 compared to control. ††† *p* < 0.001 compared to LBT alone.

Labetalol (2.5 × 10^−4^ M) inhibited the migration of H9c2 rat cardiomyoblasts (*p* < 0.001 compared to control, Figure 3). However, lipid emulsion (1%) partially restored the reduction in cell migration induced by labetalol (2.5 × 10^−4^ M) (*p* < 0.001 compared to labetalol alone, Figure 3).

**Figure 4:**
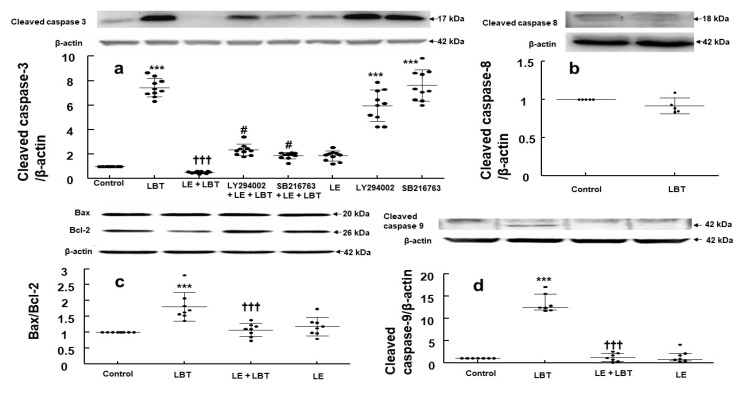
(**a**) Effect of labetalol (LBT, 2.5 × 10^−4^ M), lipid emulsion (LE, 1%), LY294002 (10^−6^ M), and SB216763 (5 × 10^−6^ M), used alone or in combination, on the expression of cleaved caspase-3 in H9c2 rat cardiomyoblasts. (**b**–**d**) Effect of LBT (2.5 × 10^−4^ M) and LE (1%), used alone or in combination, on the expression of cleaved caspase-8 (**b**), Bax/Bcl-2 ratio (**c**), and cleaved caspase-9 (**d**). Data ((**a**,**c**,**d**): *N* = 5; (**b**): *N* = 4) are presented as mean ± SD (**a**–**c**) or median ± interquartile range (25–75%, (**d**)). *N* indicates the number of separate experiments. *** *p* < 0.001 compared to control. ††† *p* < 0.001 compared to LBT alone. # *p* < 0.001 compared to LE + LBT.

Labetalol (2.5 × 10^−4^ M) elevated the cleaved caspase-3 expression in H9c2 rat cardiomyoblasts (*p* < 0.001 compared to control, Figure 4a). This increase was reduced by lipid emulsion (1%) (*p* < 0.001 compared to labetalol alone, Figure 4a). The PI3K inhibitor LY294002 (10^−6^ M) and the GSK-3β inhibitor SB216763 (5 × 10^−6^ M) partially reversed the lipid emulsion (1%)-mediated suppression of this effect of labetalol (*p* < 0.001 compared to lipid emulsion plus labetalol, Figure 4a). Labetalol (2.5 × 10^−4^ M) did not significantly alter the cleaved caspase-8 expression (Figure 4b). However, it increased the Bax/Bcl-2 expression ratio (*p* < 0.001 compared to control, Figure 4c), whereas lipid emulsion (1%) alleviated this increase (*p* < 0.001 compared to labetalol alone, Figure 4c). Additionally, labetalol (2.5 × 10^−4^ M) increased the cleaved caspase-9 expression (*p* < 0.001 compared to control, Figure 4d), and this effect was reversed by the lipid emulsion (1%) (*p* < 0.001 compared to labetalol alone, Figure 4d).

**Figure 5:**
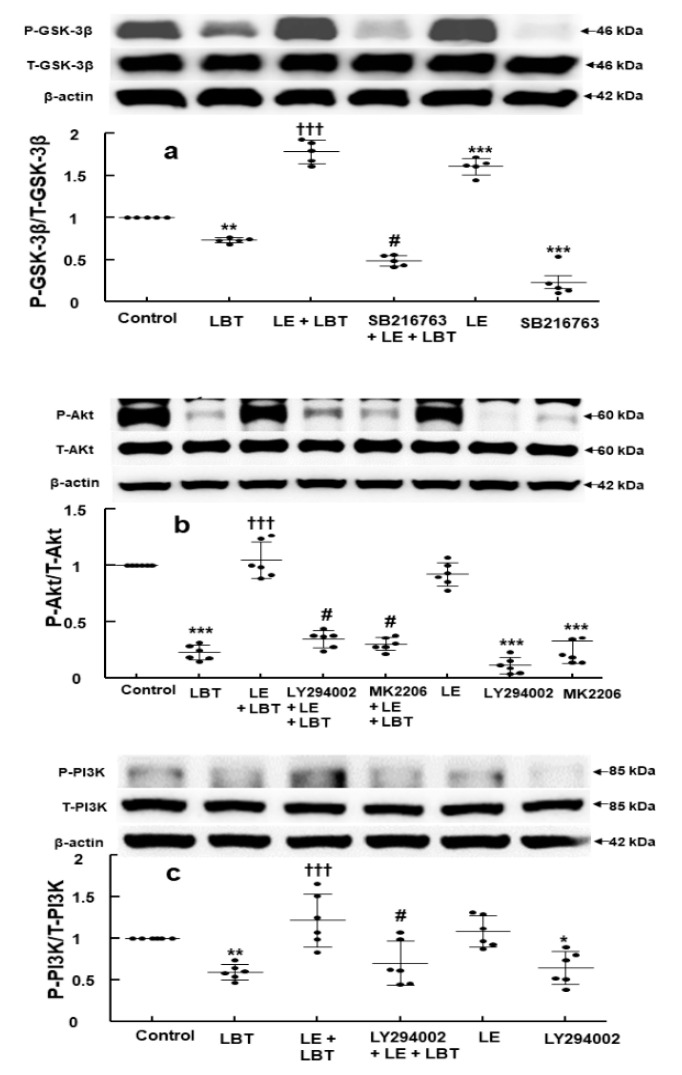
(**a**) Effect of labetalol (LBT, 2.5 × 10^−4^ M), lipid emulsion (LE, 1%), and SB216763 (5 × 10^−6^ M), used alone or in combination, on the phosphorylation of glycogen synthase kinase-3β (GSK-3β) in H9c2 rat cardiomyoblasts. Data (*N* = 4) are expressed as mean ± SD. ** *p* < 0.01 and *** *p* < 0.001 compared to control. ††† *p* < 0.001 compared to LBT alone. # *p* < 0.001 compared to LE + LBT. P-GSK-3β: phosphorylated GSK-3β; T-GSK-3β: total GSK-3β. (**b**) Effect of LBT, LE, LY294002 (10^−5^ M), and MK2206 (10^−7^ M), used alone or in combination, on Akt phosphorylation in rat cardiomyoblasts. Data (*N* = 4) are presented as mean ± SD. *** *p* < 0.001 compared to control. ††† *p* < 0.001 compared to LBT alone. # *p* < 0.001 compared to LE + LBT. P-Akt: phosphorylated Akt; T-Akt: total Akt. (**c**) Effect of LBT, LE, and LY294002 (10^−5^ M), used alone or in combination, on the phosphorylation of phosphoinositide-3 kinase (PI3K) in rat cardiomyoblasts. Data (*N* = 6) are expressed as mean ± SD. *N* represents the number of separate experiments. * *p* < 0.05 and ** *p* < 0.01 compared to control. ††† *p* < 0.001 compared to LBT alone. # *p* < 0.001 compared to LE + LBT. P-PI3K: phosphorylated PI3K; T-PI3K: total PI3K.

Labetalol (2.5 × 10^−4^ M) reduced the phosphorylation of GSK-3β (*p* < 0.01 compared to control, Figure 5a), but lipid emulsion (1%) restored it (*p* < 0.001 compared to labetalol alone, Figure 5a). The GSK-3β inhibitor SB216763 (5 × 10^−6^ M) diminished this lipid emulsion (1%)-mediated recovery of GSK-3β phosphorylation (*p* < 0.001 compared to lipid emulsion with labetalol, Figure 5a). Additionally, lipid emulsion (1%) alone increased GSK-3β phosphorylation (*p* < 0.001 compared to control, Figure 5a). Labetalol (2.5 × 10^−4^ M) also decreased the phosphorylation of Akt (*p* < 0.001 compared to control, Figure 5b), but lipid emulsion (1%) reversed it (*p* < 0.001 compared to labetalol alone, Figure 5b). Moreover, the Akt inhibitor MK2206 (10^−7^ M) and the PI3K inhibitor LY294002 (10^−5^ M) inhibited the reversal of labetalol-induced reduction in Akt phosphorylation through lipid emulsion (*p* < 0.001 compared to lipid emulsion plus labetalol, Figure 5b). Labetalol (2.5 × 10^−4^ M) also reduced the phosphorylation of PI3K (*p* < 0.01 compared to control, Figure 5c). Lipid emulsion (1%) mitigated this reduction (*p* < 0.001 compared to labetalol alone, Figure 5c). Furthermore, the PI3K inhibitor LY294002 (10^−5^ M) reduced the lipid emulsion-mediated recovery of PI3K phosphorylation suppressed by labetalol (2.5 × 10^−4^ M) (*p* < 0.001 compared to lipid emulsion plus labetalol, Figure 5c).

**Figure 6:**
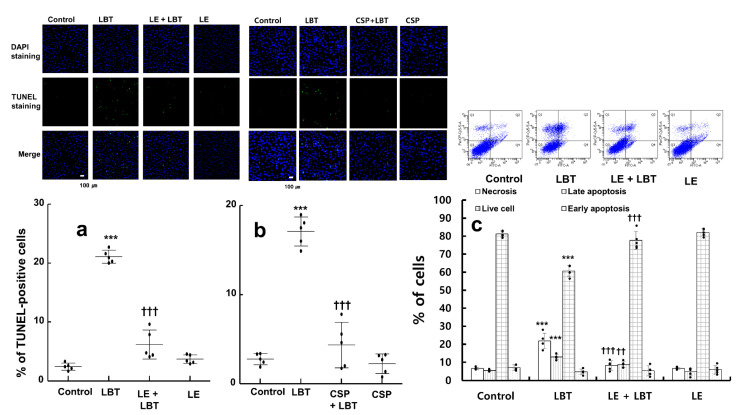
Effect of lipid emulsion (LE, 1%) or cyclosporin (CSP, 10^−8^ M) on labetalol (LBT, 2.5 × 10^−4^ M)-induced apoptosis was evaluated using TUNEL staining (**a**,**b**) and annexin V-fluorescein isothiocyanate-propidium iodide staining (**c**) in H9c2 rat cardiomyoblasts. Data (*N* = 5) are presented as mean ± SD, where *N* represents the number of separate experiments. *** *p* < 0.001 compared to control. †† *p* < 0.01 and ††† *p* < 0.001 compared to LBT alone.

Labetalol (2.5 × 10^−4^ M) significantly elevated the number of TUNEL-positive H9c2 rat cardiomyoblasts (*p* < 0.001 compared to control, Figure 6a). However, lipid emulsion (1%) reduced this increase (*p* < 0.001 compared to labetalol alone, Figure 6a). The MPTP inhibitor cyclosporin (10^−8^ M) also reduced the number of TUNEL-positive cells caused by labetalol (2.5 × 10^−4^ M) (*p* < 0.001 compared to labetalol alone, Figure 6b). Labetalol (2.5 × 10^−4^ M) promoted late apoptosis (*P* < 0.001 compared to control, Figure 6c), but lipid emulsion (1%) prevented this labetalol-induced promotion (*p* < 0.01 compared to labetalol alone, Figure 6c).

**Figure 7:**
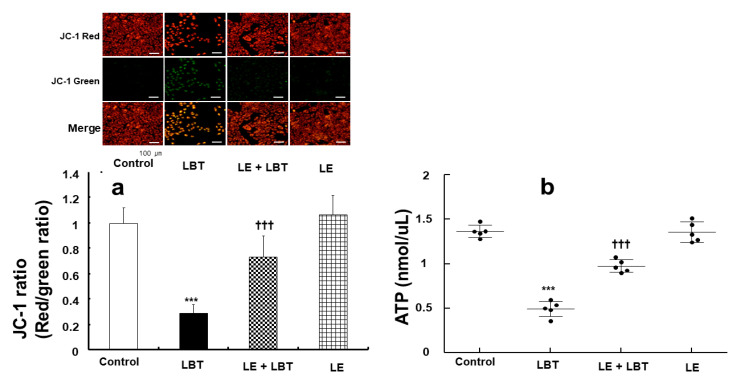
Effect of lipid emulsion (LE, 1%) and labetalol (LBT, 2.5 × 10^−4^ M), used alone or in combination, on mitochondrial membrane potential (**a**) and adenosine triphosphate (ATP, (**b**)) levels in H9c2 rat cardiomyoblasts. Data ((**a**): *N* = 3, (**b**): *N* = 5) are presented as mean ± SD, with *N* representing the number of separate experiments. *** *p* < 0.001 compared to control. ††† *p* < 0.001 compared to LBT alone.

Labetalol (2.5 × 10^−4^ M) reduced the mitochondrial membrane potential in rat cardiomyoblasts (*p* < 0.001 compared to control, Figure 7a), but this effect was mitigated by lipid emulsion (1%) (*p* < 0.001 compared to labetalol alone, Figure 7a). Labetalol (2.5 × 10^−4^ M) also decreased the ATP levels in rat cardiomyoblasts (*p* < 0.001 compared to control, Figure 7b), whereas lipid emulsion (1%) partially restored the ATP levels (*p* < 0.001 compared to labetalol alone, Figure 7b).

## 4. Discussion

This study showed that lipid emulsion mitigates late-stage apoptosis triggered by a toxic dose of labetalol by reducing the intrinsic apoptotic pathway in rat cardiomyoblasts and that this effect is likely mediated via the PI3K, Akt, and GSK-3β phosphorylation pathway. The key findings of this study were as follows: (1) Lipid emulsion alleviated the decrease in cell viability caused by a toxic dose of labetalol, and this effect was partially reversed by the Akt inhibitor MK2206, PI3K inhibitor LY294002, and GSK-3β inhibitor SB216763. (2) LY294002 and SB216763 blocked the ability of the lipid emulsion to reduce the labetalol-induced augmentation in cleaved caspase-3 expression. (3) Lipid emulsion reversed the labetalol-induced augmentation in the Bax/Bcl-2 expression ratio and the decrease in phosphorylation of GSK-3β, Akt, and PI3K. (4) Lipid emulsion prevented labetalol-induced late apoptosis, mitochondrial membrane depolarization, and ATP reduction.

Lipid emulsion alleviated the reduction in cell viability caused by a toxic dose of labetalol. Consistent with previous studies [7,8,9], the inhibitors LY294002, SB216763, and MK2206 partially blocked the effect of lipid emulsion in reversing the labetalol-induced decrease in cell viability, indicating that the restoration of cell viability by lipid emulsion in response to the toxic dose of labetalol is mediated through the PI3K, Akt, and GSK-3β pathway. In line with the cell viability findings, lipid emulsion also mitigated the decrease in cell migration caused by the toxic labetalol dose (Figure 3), further supporting the notion that lipid emulsion helps restore the impairment of both cell viability and migration by toxic labetalol levels.

The apoptosis of cells proceeds via intrinsic or extrinsic pathways, which are triggered by mitochondrial stress and death receptors, respectively, ultimately resulting in the formation of cleaved caspase-3 [18]. The intrinsic apoptotic pathway, initiated by mitochondrial stress, is regulated by proapoptotic proteins (such as Bak and Bax) and anti-apoptotic proteins (such as Bcl-XL and Bcl-2), leading to the activation of cleaved caspase-9 [18]. On the contrary, the extrinsic pathway is triggered by the activation of death receptors on the cell surface, which subsequently activate caspase-8 [18]. Lipid emulsion reduces apoptosis induced by bupivacaine or verapamil in cardiomyoblasts through the PI3K and GSK-3β or PI3K and Akt pathways, respectively [7,9]. Additionally, postischemic treatment with lipid emulsion offers cardiac protection via activation of PI3K, Akt, and GSK-3β phosphorylation [8]. Lipid emulsion reduced the labetalol-induced increase in cleaved caspase-3 expression. Consistent with the cell viability results and previous studies [7,8,9], the PI3K (LY294002) and GSK-3β (SB216763) inhibitors reduced the ability of lipid emulsion to reverse the labetalol-induced increase in cleaved caspase-3 expression, indicating that the effect of lipid emulsion is partially mediated via the PI3K and GSK-3β pathways. Labetalol increased the Bax/Bcl-2 expression ratio and the expression of cleaved caspase-9 in H9c2 rat cardiomyoblasts. However, these effects were decreased by the lipid emulsion. Labetalol did not significantly affect the expression of cleaved caspase-8. Together, these results imply that lipid emulsion inhibits the intrinsic apoptotic pathway in the mitochondria, likely via the phosphorylation of PI3K and GSK-3β. Lipid emulsion has been shown to reduce the concentration of labetalol in its aqueous phase, indicating sequestration of the drug [10,11]. Therefore, lipid emulsion-mediated reversal of the decrease in cell viability and increase in cleaved caspase-3 and Bax/Bcl-2 expression caused by labetalol may be attributed to the absorption of labetalol, which is highly lipid-soluble (log P: 2.7), by the lipid emulsion, leading to a reduction in the labetalol concentration in contact with rat cardiomyoblasts [10]. In the present study, the GSK-3β inhibitor SB216763 impaired the ability of lipid emulsion to reverse the reduction in cell viability caused by the toxic dose of labetalol. SB216763 can induce apoptosis in human osteosarcoma cells by downregulating GSK-3β expression and significantly inhibits GSK-3β phosphorylation in serum-starved retinal neurons [19,20]. Therefore, we assessed the impact of lipid emulsion and SB216763, either alone or in combination, on labetalol-induced reduction in GSK-3β phosphorylation. In line with the inhibitory effect of SB216763 on the reversal of labetalol-induced reduction in cell viability by the lipid emulsion, the lipid emulsion reversed the labetalol-induced decrease in GSK-3β phosphorylation, which likely contributed to the reduced apoptosis. However, SB216763 diminished the ability of the lipid emulsion to reverse the labetalol-induced decrease in GSK-3β phosphorylation, suggesting that this inhibition of GSK-3β phosphorylation may be linked to increased apoptosis. These findings imply that GSK-3β phosphorylation plays a role in the lipid emulsion-induced reversal of decreased cell viability caused by labetalol. Additionally, lipid emulsion reversed the labetalol-induced decrease in Akt phosphorylation, whereas inhibitors of PI3K (LY294002) and Akt (MK2206) blocked this reversal. Furthermore, lipid emulsion mitigated the reduction in PI3K phosphorylation caused by labetalol, but PI3K inhibitors inhibited this effect. Together, these results indicate that the ability of lipid emulsion to reverse labetalol-induced reduction in Akt phosphorylation is mediated via PI3K signaling. Based on previous studies [7,8,9], the reversal of labetalol-induced apoptosis by lipid emulsion is apparently driven by the involvement of the PI3K-Akt-GSK-3β pathway. However, as indicated in a previous report [11], it is challenging to completely separate this signaling pathway from the scavenging effect of lipid emulsion on labetalol. Therefore, further investigations are needed to fully understand the upstream signaling mechanisms responsible for the activation of the PI3K-Akt-GSK-3β pathway in this context. The active form of GSK-3β (non-phosphorylated) is involved in the inhibition of cellular signal pathways associated with cardiac hypertrophy [21,22]. As lipid emulsion alone increased GSK-3β phosphorylation, which may lead to enhanced cardiac hypertrophy, further examination of the effect of lipid emulsion on the hypertrophic signal pathway in the heart is needed. Caspase-3 is involved in non-apoptotic functions, which help enhance the endothelial barrier integrity [23]. In addition, cell-autonomous activation of caspase-3 is associated with the intrinsically mediated caspase-3 activation without cell death [24]. In this study, the inhibitors LY294002 and SB216763 had no effect on cell viability when used alone, but they increased the expression of cleaved caspase-3. Considering previous reports, this inhibitor-induced caspase-3 activation might be associated with the non-apoptotic function [23,24]. Further studies on casapase-3 activation induced by these inhibitors alone are needed.

In line with the reduction in cleaved caspase-3 expression induced by lipid emulsion, the emulsion also decreased the labetalol-induced enhancement in the number of TUNEL-positive cells and promoted late-stage apoptosis. The permeability of the mitochondrial membrane is regulated by the Bcl-2 family proteins [25]. The transition of mitochondrial permeability occurs when there is a rapid rise in the permeability of the inner mitochondrial membrane to solutes and water, induced by the opening of the MPTP [25]. This results in membrane depolarization and decreased ATP production [25]. The opening of MPTP was reported to block the decrease in apoptosis mediated by lipid emulsion in response to bupivacaine [9]. In this study, the MPTP inhibitor cyclosporin reduced the labetalol-induced increase in the number of TUNEL-positive cells. These findings indicate that the increase in the number of TUNEL-positive cells caused by labetalol may be mediated by the opening of the MPTP. The lipid emulsion reduced the mitochondrial membrane depolarization and decreased ATP levels caused by labetalol. Considering previous reports [7,8,9], these results indicate that lipid emulsion may inhibit the opening of the MPTP through a signaling pathway involving phosphorylated PI3K, Akt, and GSK-3β. In addition, lipid emulsion inhibited labetalol-induced necrosis (Figure 6c). Thus, further studies are needed to decipher the mechanism underlying the lipid emulsion-mediated reversal of labetalol-induced necrosis (uncontrolled cell death).

This study had several limitations. First, we utilized a rat cardiomyoblast line and not primary cultured cardiomyocytes, which would be a more appropriate model. Second, the study was conducted in vitro using rat cardiomyoblasts. In vivo experiments would be more relevant to clinical scenarios involving cardiac suppression resulting from a toxic dose of labetalol. Third, this study involved pretreatment with lipid emulsion; however, in clinical settings, lipid emulsion is typically administered after the onset of cardiac suppression caused by a toxic dose of labetalol. Despite these limitations, this study has shown that lipid emulsion mitigates cardiovascular suppression caused by toxic doses of labetalol, which does not respond to conventional supportive treatments [6]. Additionally, lipid emulsion reduces severe vasodilation induced by labetalol toxicity by inhibiting nitric oxide production [11]. Given these findings, lipid emulsion may serve as adjunctive therapy to counteract the cardiac depression and vasodilation evoked by toxic labetalol levels (toxic concentration of plasma labetalol: 1 μg/mL, 1.94 × 10^−6^ M) [15]. Moreover, when considered with previous reports, lipid emulsion might mitigate cardiac depression caused by toxic doses of lipid soluble drugs, such as verapamil and bupivacaine, which is apparently mediated by the cellular pathway involving GSK-3β, Akt, and PI3K [7,8,9]. However, further investigations on the role of this pathway in cardiac depression induced by toxic doses of drugs are needed. The use of 1% plasma triglyceride as an adjunctive treatment, which offers both positive inotropic and scavenging effects, is recommended for managing cardiovascular depression resulting from toxic doses of non-local anesthetic drugs [26,27]. Therefore, in this study, 1% lipid emulsion was used to investigate its effects on the decrease in the viability of rat cardiomyoblasts caused by toxic labetalol doses.

## 5. Conclusions

Lipid emulsion reduced apoptosis induced by a toxic dose of labetalol in rat cardiomyoblasts by suppressing the intrinsic apoptotic pathway and opening the MPTP. This effect appears to be mediated through the phosphorylated PI3K, Akt, and GSK-3β. Based on our findings, lipid emulsion may serve as an adjunctive therapy to counteract the cardiac depression and vasodilation caused by toxic labetalol levels and needs to be investigated further in future studies.

## Data Availability

The data presented in this study are available from the corresponding author upon reasonable request.
